# Proteome interrogation using gold nanoprobes to identify targets of arctigenin in fish parasites

**DOI:** 10.1186/s12951-020-00591-9

**Published:** 2020-02-18

**Authors:** Xiao Tu, Xiaoping Tan, Xiaozhou Qi, Aiguo Huang, Fei Ling, Gaoxue Wang

**Affiliations:** 1 College of Animal Science and Technology, Northwest A&F University, Yangling, 712100 People’s Republic of China; 2 School of Life Sciences, South China Normal University, Guangzhou, 510631 People’s Republic of China

**Keywords:** Gold nanoparticles, Arctigenin, Target identification, Monogenean

## Abstract

Gold nanoparticles (GNPs) are one of the most widely used nanomaterials in various fields. Especially, the unique chemical and physical properties make them as the promising candidates in drug target identification, unfortunately, little is known about their application in parasites. In this paper, GNPs were employed as new solid support to identify drug targets of natural bioactive compound arctigenin (ARG) against fish monogenean parasite *Gyrodactylus kobayashi*. Before target identification, GNPs with ARG on the surface showed the ability to enter the live parasites even the nucleus or mitochondria, which made the bound compounds capable of contacting directly with target proteins located anywhere of the parasites. At the same time, chemically modified compound remained the anthelminthic efficacy against *G. kobayashii*. The above results both provide assurance on the reliability of using GNPs for drug target-binding specificity. Subsequently, by interrogating the cellular proteome in parasite lysate, myosin-2 and UNC-89 were identified as the potential direct target proteins of ARG in *G. kobayashii*. Moreover, results of RNA-seq transcriptomics and iTRAQ proteomics indicated that myosin-2 expressions were down-regulated after ARG bath treatment both in transcript and protein levels, but for UNC-89, only in mRNA level. Myosin-2 is an important structural muscle protein expressed in helminth tegument and its identification as our target will enable further inhibitor optimization towards future drug discovery. Furthermore, our findings demonstrate the power of GNPs to be readily applied to other parasite drugs of unknown targets, facilitating more broadly therapeutic drug design in any pathogen or disease model.

## Background

Natural products have been an essential source for the discovery and development of new drugs [1–3]. Identification of their molecular targets is the focal point of interest, which allows for the thorough exploitation of their therapeutic potential and minimizes their adverse side effects [4]. Currently, a widely applied method to find active compound and target protein pairs relies on affinity chromatography [4]. In this way, bioactive compound is attached to a solid support (such as sepharose or agarose) to pull down proteins after incubation with cell lysate [5–7]. Bound proteins are separated by SDS-PAGE and then identified using MS-based proteomics approaches [8]. However, due to the large size of testing beads, it is uncertain to confirm whether the target-binding specificity is altered by chemical modification and the linkage to the solid support.

Gold nanoparticles (GNPs) have been used for many applications including drug delivery, cancer diagnostics and therapy, medical imaging and non-optical biosensors [9–18]. Besides, their unique chemical and physical properties made them as an attractive candidate for drug target identification. Small compounds with the terminal group of the thiolate ligands can be bound covalently to the surface of GNPs [19]. GNPs with small size can easily enter cells and make connected compounds capable of contacting directly with target proteins. Besides, the biological activity of chemically modified compounds on the surface of GNPs can also be detected [8]. In a recent study, α-tubulin and HSP 90 have been identified and validated as the targets of the cancer-killing thiazolidinone compounds by using GNPs. Unfortunately, studies on the application of GNPs for the target identification in other disease areas, such as parasites, need extensive investigation.

Arctigenin (ARG) is a natural dibenzylbutyrolactone lignan purified from *Arctium lappa* L that has been used in traditional Chinese medicine for treating a wide variety of diseases from rhinitis to fever for centuries [20]. Numerous studies have revealed that ARG possesses multiple biological activities, including anti-inflammatory, anti-viral, antitumor and immunomodulatory effects [20–23]. Fortuitously, our team have showed the potent and fast-acting activity of ARG against fish monogenean parasites [24, 25], which are responsible for significant economic losses in aquaculture [26–28]. The anthelmintic effects of ARG were proved to be associated with the damage of tegumental musculature [24], while the precise targets remain elusive.

**Fig. 1:**
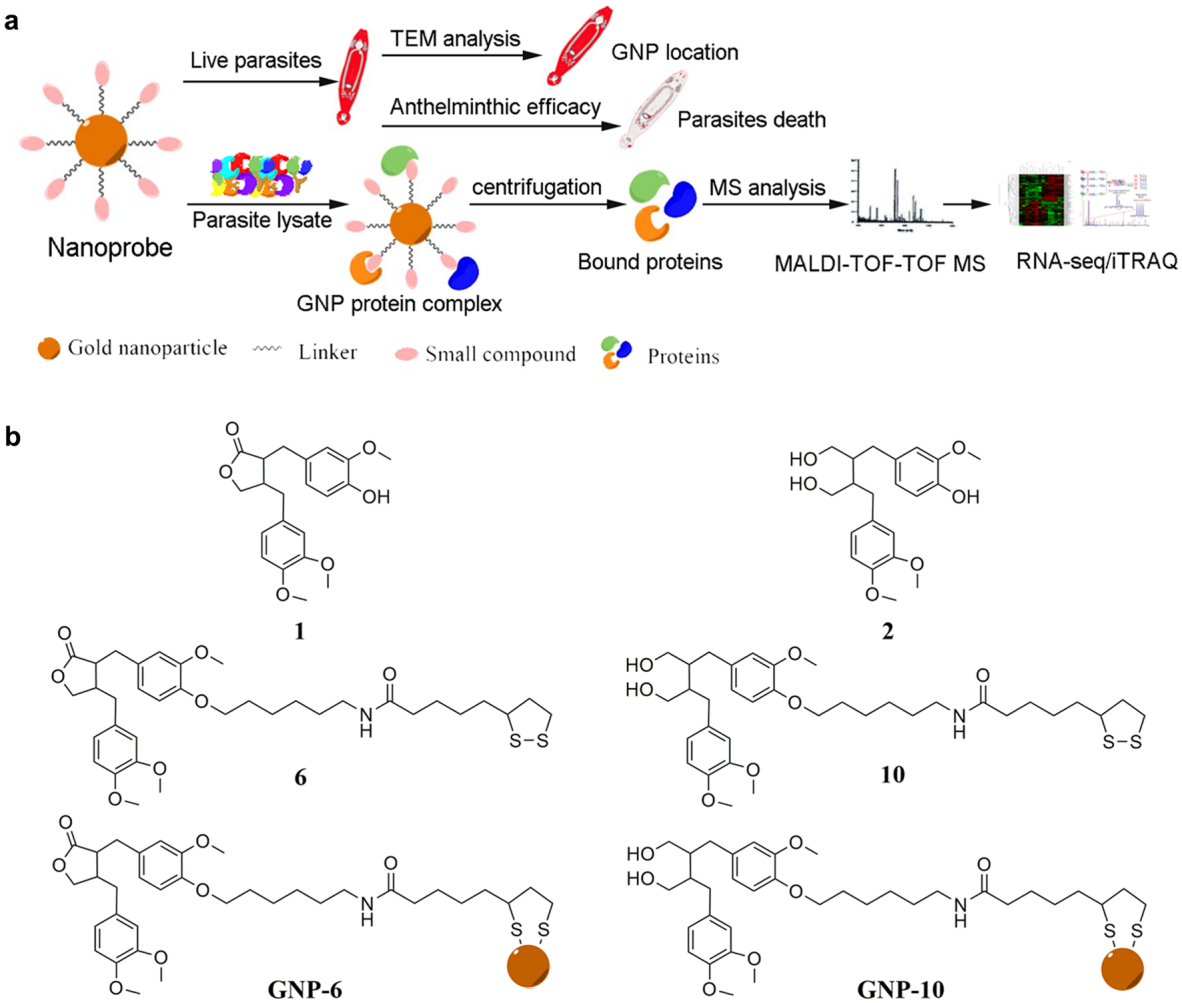
**a** Schematic presentation of drug target identification in parasites by using gold nanoprobes. **b** Structure of **1** and **2**, their derivative **6** and **10**, GNP-**6** and GNP-**10**

In this study, the possibility of GNPs as solid support in target identification was further verified in a model of *Gyrodactylus kobayashii* (Monogenea). Furthermore, the potential molecular target of ARG as myosin-2 was demonstrated by using RNA-seq transcriptomic and iTRAQ proteomic studies (Fig. 1a). The study provided a new strategy for the target identification of antiparasitic drugs and demonstrated the power of GNPs in drug discovery research.

## Methods

### Materials and parasites

ARG was extracted and purified from the traditional medicine the seeds of *A. lappa* based on our previous study [25]. Hydrogen tetrachloroaurate (III) tetrahydrate were purchased from Energy Chemistry Industry Co., Ltd. (China). Other chemicals were purchased from Sigma-Aldrich (St. Louis, MO, USA). Organic solvents were purchased from Sinopharm chemical reagent Co., Ltd and purified by distillation and moisture was excluded from the glass apparatus using CaCl_2_ drying tubes. Goldfish (8.86 ± 1.82 g) were purchased from a local fish farm in Xi'an, China and kept in indoor aerated tanks with circulating water. Goldfish-*G. kobayashii* model was established following the method as previously described [24, 29]. All goldfish were maintained in conformity with the General Recommendation of Chinese Experimental Animals Administration Legislation.

### Synthesis of compound GNP-*6* and GNP-*10*

The synthetic routes of compound **2**, **6** and **10** were shown in the Additional file 1: Figs. S1, S2, S5. The structures of all synthesized compounds were confirmed by HRMS, ^1^H, and ^13^C NMR (see the Additional file 1). 10.8 mL of water containing hydrogen tetrachloroaurate (III) tetrahydrate (132.4 mg, 0.032 mmol) was added to a solution of compound **6** or **10** (0.216 mmol) in DMF (100.0 mL). After stirring for 30 min at room temperature, sodium tetrahydroborate (34.2 mg, 0.928 mmol) in 58.8 mL water was added to the mixture dropwise. The solution turned red immediately and was stirred for another 4 h at room temperature. 1 M HCl was added to the reaction mixture dropwise to neutralize the excess sodium tetrahydroborate until the pH reached 7.0. The reaction mixture was then centrifuged at 15,000 rpm for 15 min. The supernatant was decanted and the solid was dissolved in 10 mL methanol and deionized water alternatively by sonication and centrifuged again at 15,000 rpm for 15 min. This wash-centrifugation cycle was repeated five times. After the final washing step, the nanoparticles were dried in vacuum at 50 °C for 12 h.

### High-resolution transmission electron microscope observation of GNP-*6*

GNP-**6** was suspended in DMSO. Ten microliter droplets of the sample were drop casted onto a piece of ultrathin Formvar-coated 200-mesh copper grid (Ted-pella, Inc.) and left to dry in air. Transmission electron microscopy (TEM) images were acquired on FEI Tecnai G2 F30 S-TWIN.

### FTIR study

Fourier transform infrared (FTIR) spectra were recorded using a Bruker Vetex70 spectrophotometer (Germany) in the wave number range of 575–4141 cm^−1^. Prior to each experiment, GNP-**6** or GNP-**10** (∼ 20 mg) was pretreated in He stream (50 mL min^−1^) at 400 °C for 1 h to remove the moisture, and then cooled to the desired temperature. The test sample was mixed with potassium bromide (KBr, 1:1000), powdered and then pressed into a small tablet. After preparation, the pellets were analyzed immediately, and the spectra were recorded by four scans with 2 cm^−1^ resolution.

### XPS analysis

X-ray photoelectron spectroscopy (XPS) measurements were carried out on an Escalab 250Xi system (Thermo Scientific), with monochromatized Al Kα radiation (1486.6 eV) at 14 kV and 20 mA. The freeze-dried sample was mounted on an Au-plated stainless steel sample holder. The XPS spectra were background subtracted by using the Shirley method and deconvoluted by using a mixed Gaussian/Lorentzian peak shape with XPSPeak software (version 4.1).

### *Quantification of ligand contents by I*_*2*_* cleavage and HPLC method*

GNP-**6** or GNP-**10** (2 mg) were dispersed in 300 µL MeOH. A solution of I_2_ (300 µL, 13 mg/mL) was added and the mixture was shaken for 30 min at room temperature. Then the naked GNPs were obtained by centrifugation at 13,000 rpm for 20 min. 400 µL of the supernatant was acquired for HPLC analysis. Meanwhile, 0.5 mM, 1.0 mM and 2.0 mM compound 6 or compound 10 were used to construct a standard curve. The samples and standards were filtrated using 0.22-mm filters, and then analyzed with a Waters 600E-2487 HPLC system using C18 column with a flow rate 1.0 mL/min. The injected sample volume was 10 μL. Data were acquired with a UV detector at 280 nm.

### Anthelmintic activity

In vivo anthelmintic activity of compound **2** was determined according to the methods described in our previous study [24]. Goldfish with medium intensity (40–60) of *G. kobayashii* were chosen for the following test. Three randomly selected fish were placed in some 5 L tanks containing 2.0 L of test solution at 24.5 ± 0.5 °C for 48 h exposure. 0.2% DMSO were used as the positive control. The number of gyrodactylids on both sides of the caudal fin was counted under a stereomicroscope in 0, 24 h and 48 h post treatment. Anthelmintic efficacy of compound **2** was calculated according to the methods of Zhou et al. [30].

In vitro assays were performed to detect the anthelmintic activity of GNP-**6** and GNP-**10**. Heavily parasitized goldfish were selected, and caudal fins were clipped and cut into small pieces. Each fin clip (50–100 parasites) were transferred individually, using watchmaker's forceps, to a well of 24-well plate containing 0.5 mL filtered tank water. GNP-**6** or GNP-**10** were then added to each well at 2.5, 5.0, 10.0, 20.0 and 50.0 μg/mL and time was defined as zero. The number of alive parasites was counted under a stereomicroscope in 0 and 1 h post treatment. Anthelmintic efficacy was calculated using the following formula: Anthelmintic efficacy = (N_hour 0_ − N_treatment_) / N_hour 0_ × 100%.

## TEM observation of parasite uptake

The method was used as described previously [24]. Heavily parasitized goldfish were chosen, caudal fins with parasites were clipped and transferred to wells of a 6-well plate with 2 mL filtered tank water. After exposure to 10, 20 and 50 μg/mL GNP-**6** for 0.5 h, fins were washed three times with PBS and fixed in 3.0% glutaraldehyde at 4 °C overnight. Specimens of parasites were isolated according to procedure detailed by Paladini et al. [31]. Parasites were post fixed for 1 h in 1% osmium tetraoxide and rinsed in PBS for three times. Then dehydrated with a series of ethanol solutions (30%, 50%, 70%, 80%, 90% and 100%) and embedded with white glues. The thin sections were cut on a using a Leica EM UC7 ultramicrotome and images were taken on a TEM-HT7700.

### Worm collection and preparation of parasite extracts

The collection of *G. kobayashii* (approximately 10,000) were obtained from goldfish of severe parasitism. Caudal fin with parasites were clipped and transferred individually to several 10 mL centrifuge tube containing 3 mL 0.65% saline. Briefly vortex the tube and let sit at room temperature for 3–5 min, discard the supernatant. Parasites were harvested and washed with 0.65% saline, and then diluted in RIPA solution (weak) supplemented with complete protease inhibitor cocktail. The soluble fraction (lysate) was extracted by ultrasonic (Sonics, VC130; 30 s on/off cycle for 5 min) and separated from the cell debris by centrifugation at 12,000 rpm for 15 min at 4 °C.

### Identification of target proteins

0.2 mM of GNP-**6** or GNP-**10** were dispersed in 300 µL of RIPA digestion solution (weak), and then mixed with 300 µL of parasite lysate in the presence or absence of 5 mM ARG, before incubation with end-over-end agitation at 4 °C for 1 h [8, 32]. After that, the GNPs with bound proteins were obtained by centrifugation at 13,000 rpm for 15 min at 4 °C. The GNPs-proteins were then washed five times with RIPA digestion solution (weak) to remove non-bound proteins. Finally, interacting proteins on GNPs were eluted with 100 µL RIPA digestion solution (strong) and separated on 12.5% SDS-PAGE followed by coomassie brilliant blue G-250 staining. Specific protein bands were excised and identified by MALDI-TOF/TOF MS. The Mascot search engine (https://www.matrixscience.com/) was used for protein identification with tandem mass spectrometry (MS/MS) by searching in Uniprot databases of *Caenorhabditis elegans*.

### RNA-sequencing, transcriptome assembly, and analyses

*Gyrodactylus kobayashii* were collected from the caudal fin of goldfish after treatment with 1.85 mg/L ARG (EC_50_ after 12 h of incubation) and 0.1% DMSO for 4 h. The fish were anesthetized with 0.02% MS222, live worms were collected and washed according to the aforementioned methods. Three independent biological replicates from different goldfish were collected on both occasions. Total RNA was extracted using an RNAprep pure Micro Kit (Tiangen Inc., Beijing, China). cDNA libraries were built by using NEBNext® Ultra™ RNA LibraryPrep Kit for Illumina® (NEB, Ipswich, MA, USA) and paired-end sequenced using the Illumina HiSeq 2500 platform at Novogene Company. The quality of the raw reads was assessed using the FastQC software v0.11.5. Raw reads of FASTQ format were processed by in-house Perl scripts to obtain clean reads by removing those containing adapters or ploy-N and low-quality reads. At the same time, Q20, Q30, GC-content and sequence duplication level of the clean data were calculated. Sequence reads were aligned to the goldfish reference genome using HISAT2 20 with default parameters to exclude the reads from the host. Retained reads were supposed to be derived from parasites and assembled using trinity software as described for de novo transcriptome assembly without a reference genome. Functional annotations and classifications were performed by using Blast2GO [33] and WEGO [34] (E value < 10^−5^), respectively. Only the unigenes related to those genes of the species from Platyhelminthes were selected for further analysis. The gene expression level is calculated by using RPKM method (Reads Per kb per Million reads) [35] and the edgeR package was used to identify differentially expressed genes (DEGs) across samples. A false-discovery-rate of ≤ 0.05 was used as a threshold for significance [36, 37]. Gene Ontology (GO) analysis was conducted for the functional classification of DEGs, and pathway analysis was carried out using Kyoto Encyclopedia of Genes and Genomes (KEGG). All expression data statistic and visualization were conduction with R package.

### iTRAQ proteomic analysis

*G. kobayashii* were treated in vivo with 4 mg/L ARG or 0.1% DMSO for 0.5 h. Worms were collected and homogenized in 200 μL of STD buffer (4%SDS, 1 mM DTT, 0.5 mM PMSF, and pH 8.0 150 mM Tris–HCl). The lysate was sonicated and then boiled for 15 min, followed by centrifugation at 14,000 g for 45 min. Protein concentration and quality were determined using the BCA Protein Assay Kit (Thermo Scientific, USA) and confirmed by SDS-PAGE. The method of digestion was given in Lv et al. [38], briefly, total protein (200 μg) taken from each sample was incubated overnight with trypsin buffer (2 μg trypsin in 40 μL of dissolution buffer) at 37 °C.

After trypsin digestion and desiccation, the peptides were labeled with 4-plex iTRAQ reagent following the manufacturer’s instructions (Applied Biosystems, Foster City, CA, USA). iTRAQ labeling reagents 113 and 114 were used to label the 2 DMSO-treated control samples, while reagents 115 and 116 to label technical duplicates of ARG-treated samples. The labeled peptides were combined and dried in vacuum. The dried peptide mixtures were subject to iTRAQ labeling, SCX fractionation and LC–MS/MS analysis. Data acquisition was performed with a Q Exactive mass spectrometer (Thermo Finnigan). The raw MS/MS data were transformed into MGF files with Proteome Discovery 1.2 (Thermo Fisher Scientific, USA) and analyzed using Mascot search engine (version 2.3.2; Matrix Science, UK). Mascot database was set up for protein identification using *G. kobayashii* reference transcriptome. The parameters for database searching were set as following: the mass tolerance was set as 0.02 Da for MS/MS and 10 ppm for MS, trypsin enzyme specificity and two max-missed cleavages were allowed, and the fixed modification included carbamidomethylation. To reduce the probability of false peptide identification, all data were reported based on a 95% confidence and FDR less than 1%. For quantitative analysis, the protein ratios are calculated as the median of only unique peptides of the protein. Proteins with fold change in a comparison > 1.2 or < 0.83 and unadjusted significance level p < 0.05 were considered differentially expressed. The GO and KEGG annotations of these proteins was performed in a manner similar to the transcripts.

## Results

### Biological evaluation of compound *2*

One of the common problems encountered in target identification using molecular probes is that the presence of linker and tag moieties may cause nonspecific adsorption. “Invalid compound” is the best choice for the reduction of nonspecific binding proteins. To create an optimal negative control for future target isolation trials, an inactive derivative of ARG (compound **2**, Fig. 1b) was obtained according to our previous structure–activity relationship studies [39]. In vivo antiparasitic efficacy of compound **2** against *G. kobayashii* were shown in Additional file 2: Fig. S8. No anthelmintic activity was found after in vivo treatment with 12.5 mg/L compound **2**, while 4.0 mg/L ARG displayed 100% efficacy against *G. kobayashii*. The EC_50_ value of compound **2** after 24 h exposure was 68.9 mg/L, which is much higher than that of ARG (compound **1**, 1.85 mg/L).

### Characterization of GNPs

The preliminary structure–activity relationship studies revealed that the ARG-imidazole hybrids compounds fully retained the biological activity [39], which suggested that a flexible and biocompatible linker could be attached to this phenolic hydroxyl group to give compound **6** (see Additional file 1: Fig. S2). Similarly, the inactive analogue **2** was modified to give compound **10**. GNP-**6** and GNP-**10** (Fig. 1b) were then synthesized in situ following the reported methods [40] (see detailed syntheses in Additional file 1). GNP-**10** derived from inactive compound **2** was used as a control (Fig. 1b) for target validation.

**Fig. 2:**
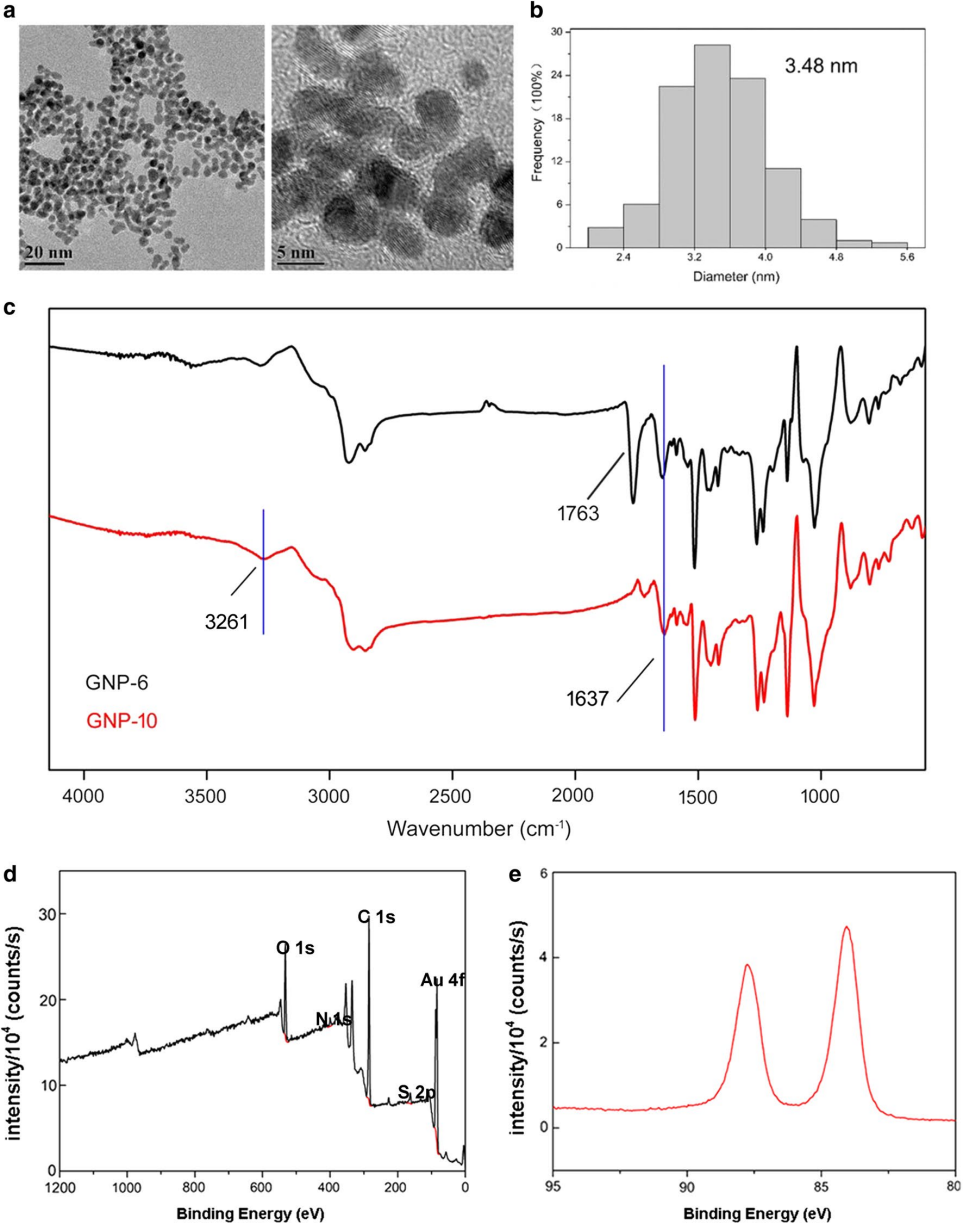
**a** TEM image of GNP-**6**. **b** Size distribution of GNP-**6** according to TEM images. The average diameter was 3.48 nm. **c** FTIR spectrum of GNPs. **d** High-resolution XPS survey spectra showing wide scan of GNP-**6**. **e** XPS region spectra of Au 4f_7/2_ and Au 4f_5/2_

As shown in high-resolution transmission electron microscopy (TEM), the average diameter of GNP-**6** particles was 3.48 nm (Fig. 2a, b). FTIR spectra for GNP-**6** and GNP-**10** are presented in Fig. 2c. The band at 1026 cm^−1^ corresponding to C–N stretching vibrations of amine and 1637 cm^−1^ corresponding to C=O stretching of amide band were both shown in GNP-**6** and GNP-**10** [41]. While the weaker band at 1763 cm^−1^ assigned to C=O stretching vibrations in lactone ring was only seen in GNP-**6**. And a weak and broad -OH stretch peak at 3261 cm^−1^ was only observed in GNP-**10**. Moreover, the chemical and electronic structure of GNP-**6** was further analyzed by XPS analysis. As shown in Fig. 2d, e, the Au4f7/2 component at 84.1 eV can be associated to the Au atoms that are covalently bonded to sulfur terminal groups of compound **6** [42]. The S 2p doublets (S 2p3/2, 1/2) observed around 162.2 and 163.3 eV of GNP-**6** were assigned to sulfur bound to gold nanoparticles [43]. FTIR and XPS studies clearly indicated that compounds **6** and **10** have been bonded successfully to the surface of GNPs.

**Fig. 3:**
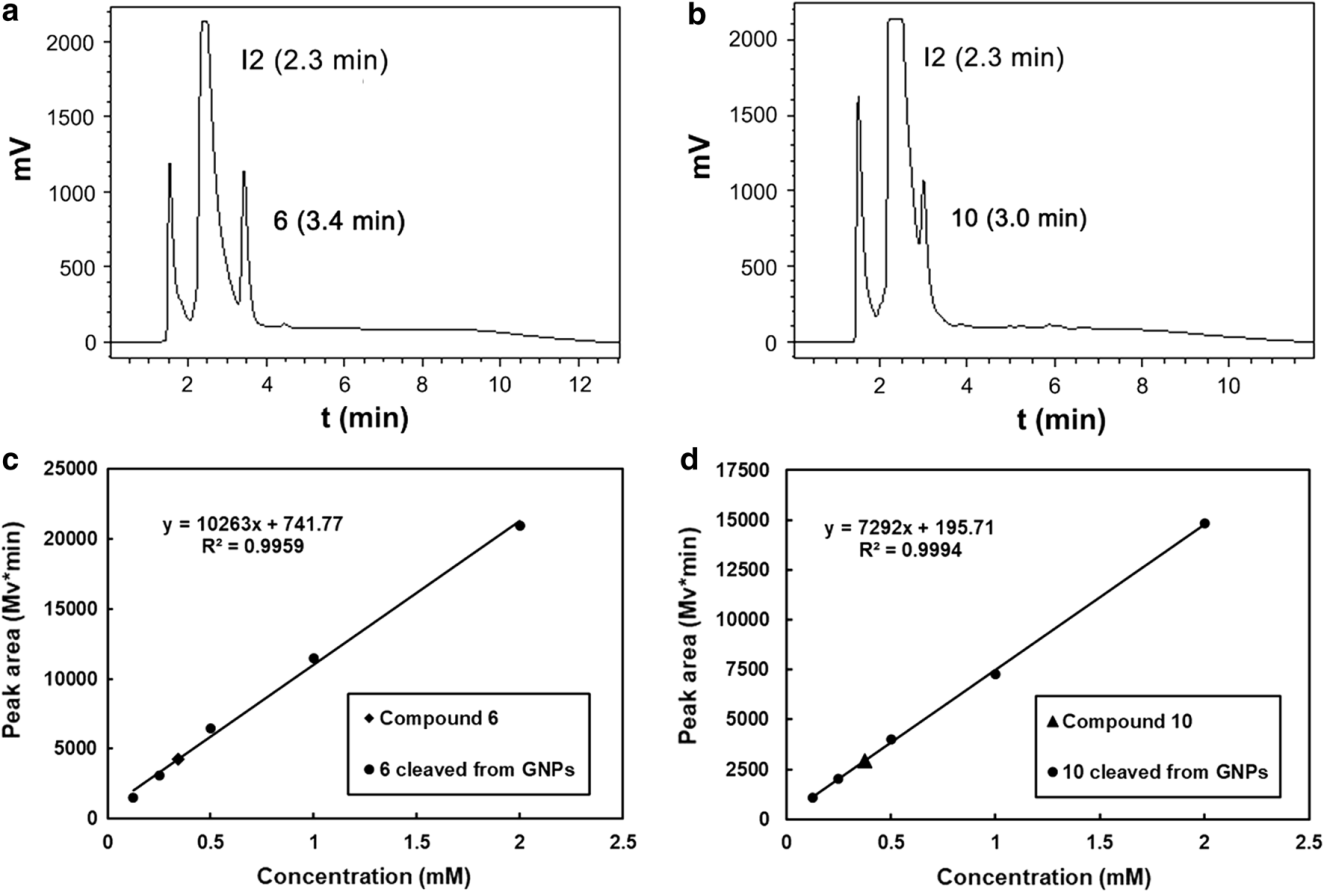
Chromatograms of free ligands cleaved from GNPs by I_2_. **a** GNP-**6**, **b** GNP-**10**. The peak at 2.3 min is I_2_, the peak at 3.4 min is compound **6**, and the peak at 3.0 min is compound **10**. The standard curves were constructed by the calculating peak areas of compound **6** (**c**) or compound **10** (**d**) in different concentrations: 0.25 mM, 0.5 mM, 1.0 mM and 2.0 mM

The quantification of each ligand on GNP-**6** or GNP-**10** were then confirmed by using I_2_ cleavage and HPLC analysis [41]. The peak at 2.3 min is I_2_, the peak at 3.4 and 3.0 min was compound **6** and **10**, respectively (Fig. 3a, b). According to the standard curve constructed by the calculating peak areas of compound **6** or **10** in different concentrations (Fig. 3c, d), the amount of **6** on the surface of GNP-**6** was 0.506 mmol/g and **10** on GNP-**10** was 0.461 mmol/g. Based on the diameter of GNPs (3.48 nm), it can be estimated that there are 198 of compound **6** on each GNP-**6** and 173 of compound **10** on each GNP-**10** [44].

### Parasite uptake and biological evaluations of GNPs

**Fig. 4:**
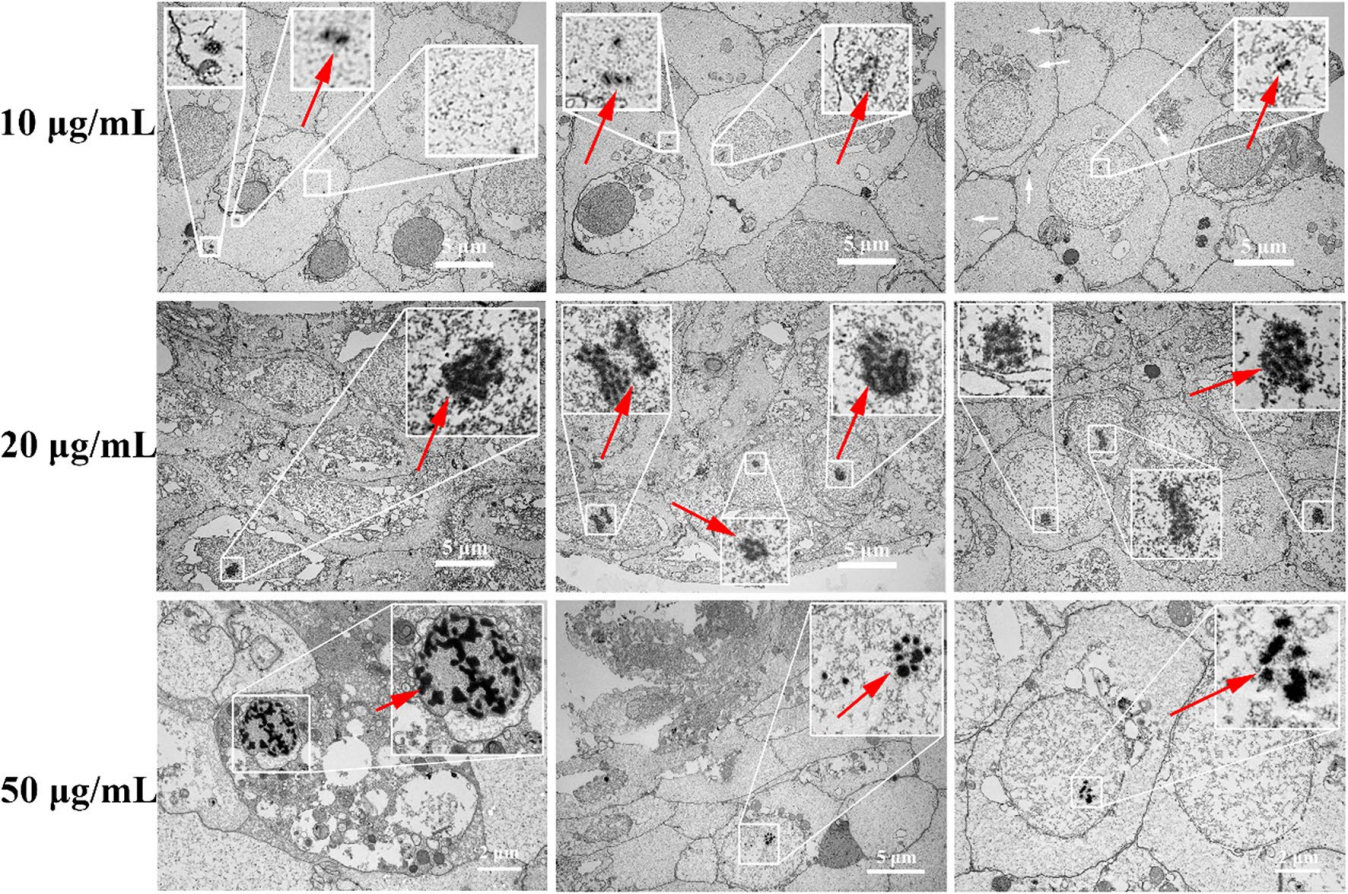
TEM images of *G. kobayashii* treated in vitro with 10, 20 and 50 μg/mL GNP-**6** (red arrows) for 0.5 h

**Fig. 5:**
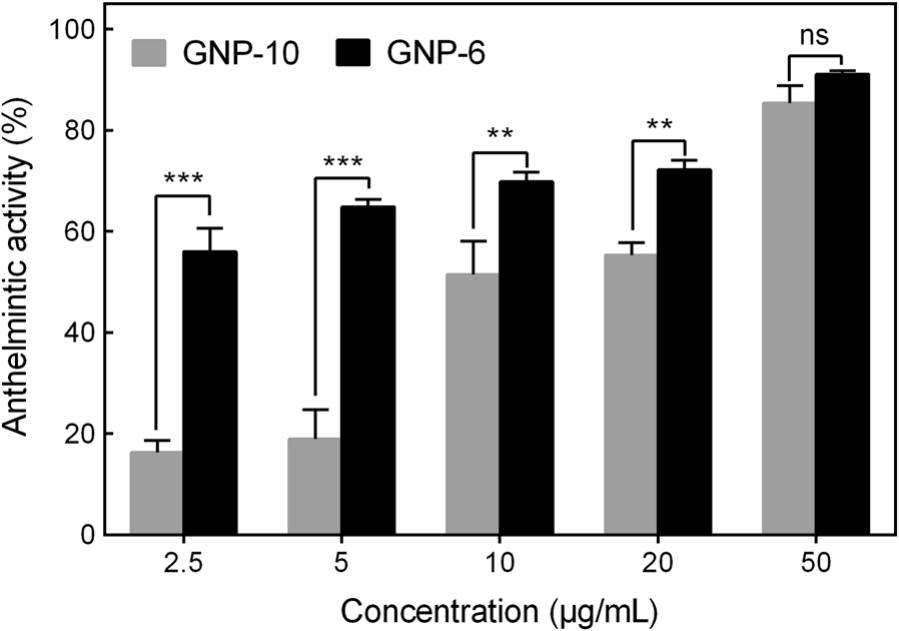
In vitro anthelmintic efficacy of GNP-**6** and GNP-**10** against *G. kobayashii* during the 0.5 h-treatment*.* Results represent mean ± SD of three independent experiments. Significance between the GNP-**6** treated groups and GNP-**10** treated groups are indicated by **P < 0.01, ***P < 0.001

The ability to enter cells and maintain the original activity of the compound-binding matrix are of great importance for improving the target binding specificity. To verify whether GNP-**6** could enter parasites, live worms were treated with 10, 20 and 50 μg/mL GNP-**6** in vitro for 0.5 h and examined by TEM. The results indicated that GNP-**6** particles could readily enter parasites, and the accumulation of nanoparticles is concentration dependent, with largest amounts found in worms treated with 50 μg/mL GNP-**6** (shown in Fig. 4). Interestingly, 10 μg/mL GNP-**6** were sparsely distributed throughout the cytoplasm, while larger clusters of nanoparticles were found in the nucleus of 50 μg/mL GNP-**6** treated worms. To determine the chemical modification and the attachment to GNPs did not alter the biological activity of compound **1**, in vitro anthelmintic activity of GNP-**6** and GNP-**10** were evaluated against *G. kobayashii*. Figure 5 showed that GNP-**6** had concentration‐dependent efficacy for *G. kobayashii* treatment, 5 μg/mL could cause 64.9% mortality. When the dosages were ≤ 20 μg/mL, anthelmintic effect of GNP-**6** was significantly higher than that of GNP-**10**. Besides, no remarkable difference was found between the treatment groups of 50 μg/mL GNP-**6** and GNP-**10**, high toxicity to parasites could be ascribed to GNPs. These results indicated that GNP-**6** retained the ability to kill parasites effectively.

### ARG directly targets myosin-2 and UNC-89

**Fig. 6:**
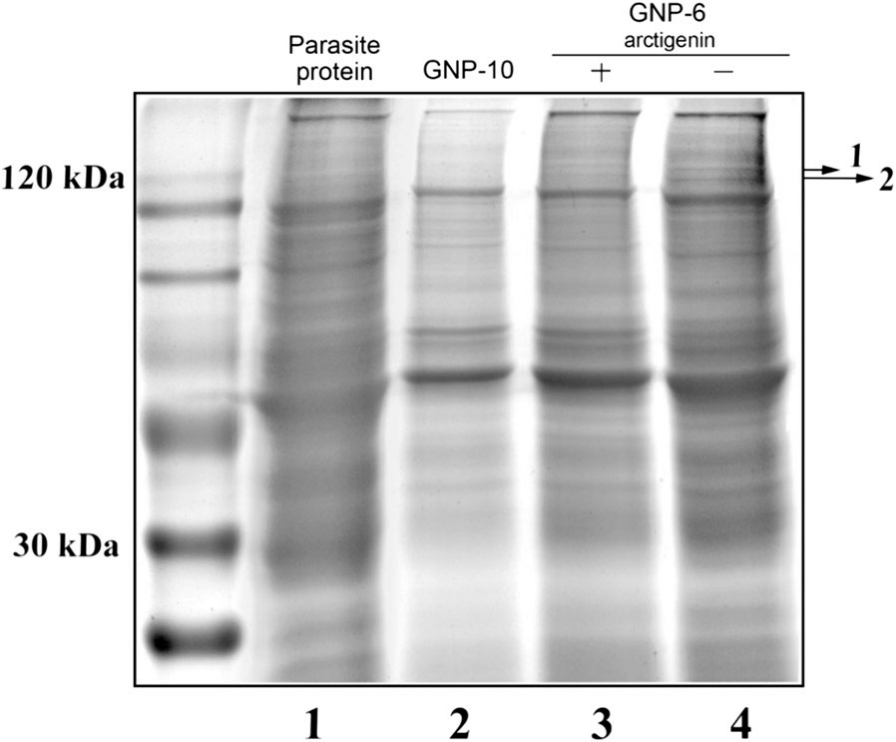
Target identification by using GNP-**6**. *G. kobayashii* cell lysate (300 µL) was incubated with (lane 3) or without (lane 4) 5 mM ARG for 1 h at 4 °C, after which 0.06 μmol of GNP-**6** was added and the mixture incubated for 1 h at 4 °C. As a control, parasite extract (300 μL) was also incubated with GNP-**10** and treated in the same way (lane 2). Proteins bound to GNPs were separated by 12.5% SDS-PAGE followed by improved Coomassie brilliant blue G-250 staining. Protein bands with lower intensities in the lane with ARG than in the lane without ARG were identified by MALDI-TOF/TOF MS and Mascot analysis (Table 1)

To identify cellular proteins that interact with ARG, GNP-**6** and GNP-**10** were incubated with parasite lysate for 1 h at 4 °C, and then the bound proteins were separated and identified by SDS-PAGE electrophoresis. More than 20 protein bands have been observed for GNP-**6** (line 4), while fewer proteins were found in GNP-**10** (line 2). We focused on the proteins only existed in line 4, two bands were shown (Fig. 6), suggesting they might be specific to ARG. To ensure that only proteins with specific binding to GNP-**6** were correctly identified, we also preincubated ARG with the lysate for 1 h at 4 °C and then incubated this lysate with GNP-**6** for an additional 1 h (lane 3). The two protein bands also displayed reduced intensities (Fig. 6) when comparing line 4 with lines 2 and 3, which indicated they were the specific target proteins of ARG. Target protein bands were cut and identified by mass spectrometry MALDI-TOF/TOF. All the MS/MS spectra were evaluated by Mascot database search (Table 1). Success of identification was based on a criterion of the protein score confidence interval (CI%) exceeding 95%. These proteins were myosin-2 and muscle M-line assembly protein UNC-89.

**Table 1:** Protein identification by MALDI-TOF/TOF MS and Mascot analysis

Band	Protein identified	Mascot score	Protein score CI (%)
1	Myosin-2	58	95.207
2	Muscle M-line assembly protein, UNC-89	69	99.676

### ARG down-regulated the transcription of myosin-2 and unc-89

**Fig. 7:**
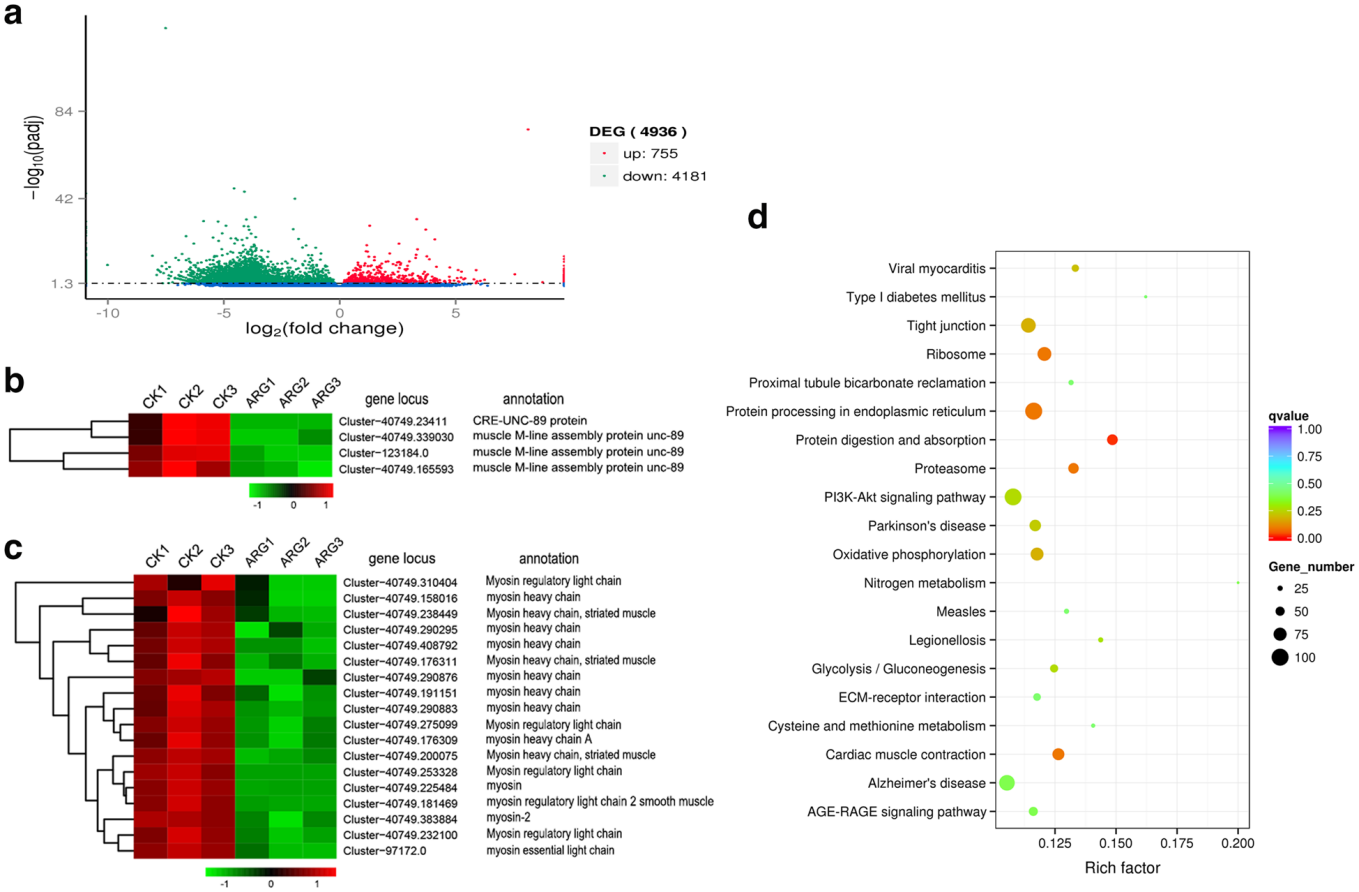
**a** Volcano plot of all differentially expressed transcripts. Significantly differentially expressed genes are shown as red (up) or green (down) dots. No significant difference is indicated by blue dots. The x-axis is the fold difference (log 2) between groups and the y-axis represents the log10 of the p-value. Heat map illustrates results from RNA-seq for UNC-89 (**b**) and myosin-2 (**c**) in ARG treated versus control parasites. Data were scaled across rows before mapping to colors (**d**). Top 20 significantly enriched KEGG pathways. The x-axis shows the enrichment factor; the y-axis corresponds to KEGG Pathway

To investigate the molecular mechanism of ARG against *G. kobayashii*, RNA-seq transcriptomic analysis was performed in 1.85 mg/L (EC_50_) ARG and DMSO treated worms. 4-h time-point was selected for the reason that 1.85 mg/L ARG showed no significant reduction in motility as well as the number of alive worms. Due to the absence of reference genomic sequences, a de novo RNA-seq assembly was performed using Trinity which produced 1,142,515 transcripts. The RNA-seq raw reads in this article have been deposited as project number SRP156825 in the NCBI Short Read Archive. Pairwise comparisons were performed on mRNA isolated from worms for ARG exposure group to allow the identification of DEGs with FDR < 0.05, relative to control. 4936 were identified as differentially transcribed in response to ARG treatment, 755 genes were up-regulated and 4181 genes were down-regulated relative to the control (Fig. 7a). Among which, the transcripts of 4 genes encoding muscle M-line assembly protein UNC-89 were dramatically down-regulated in respose to ARG exposure (Fig. 7b). Additionally, 18 genes encoding moysin-2 including myosin regulatory light chain and myosin heavy chain were also generally down-regulated by ARG exposure. The significantly DEGs were then subjected to a KEGG pathways analysis using DAVID software, the top 20 enriched pathways were presented in Fig. 7d. We noted that the pathways “cardiac muscle contraction”, “ECM-receptor interaction” and “tight junction” were significantly enriched (*p * value < 0.01), suggesting that ARG exposure elicits negative consequence on the tegument of worms.

### Identification of DEPs through iTRAQ

**Fig. 8:**
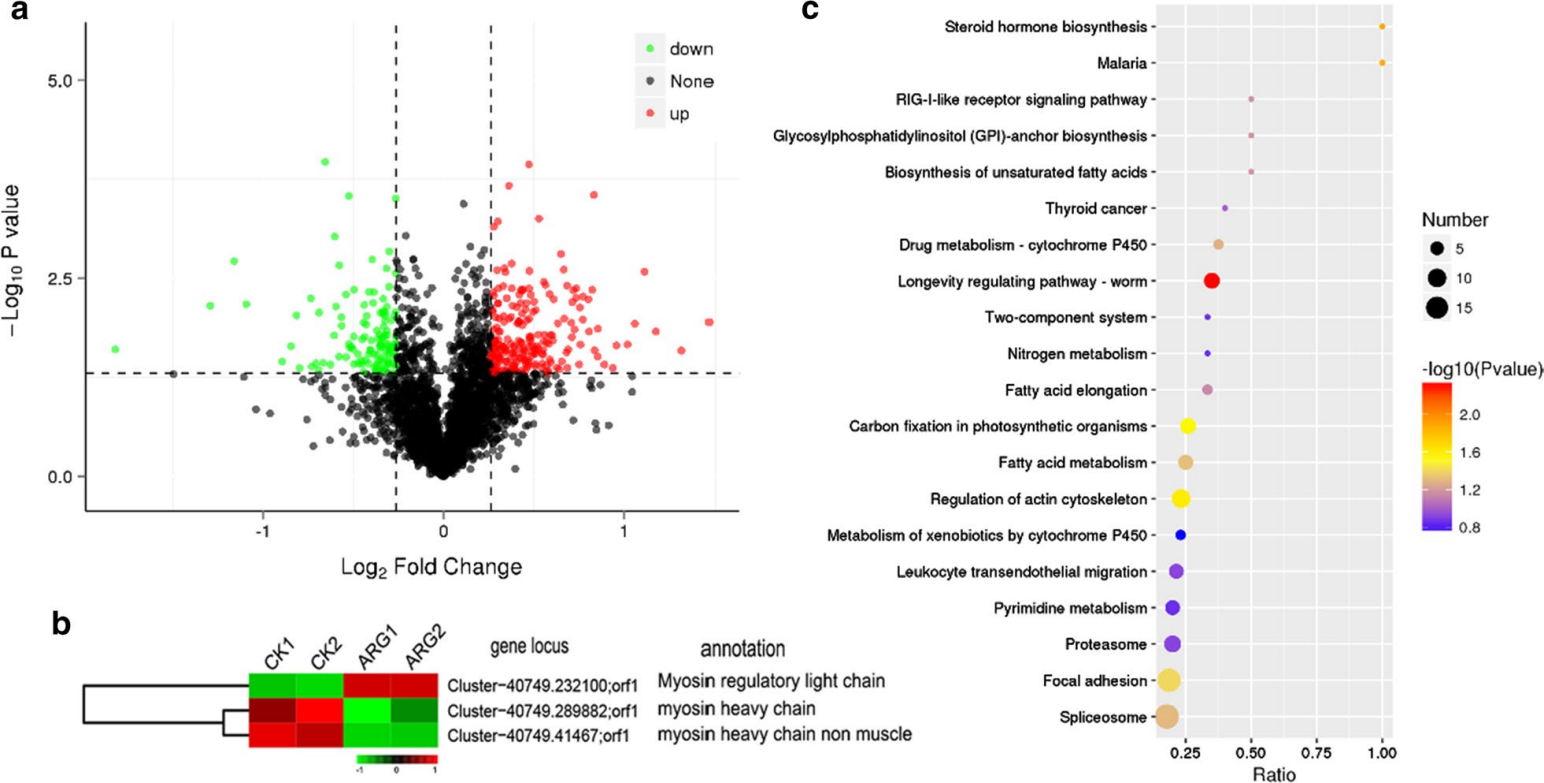
**a** Ratio distribution of all quantitative proteins. Differentially expressed protein marked in red (up) or green (down). No significant difference is indicated by black dots. **b** Heat map illustrates results from iTRAQ for myosin in ARG treated versus control parasites. Data were scaled across rows before mapping to colors. **c** Top 20 significantly enriched KEGG pathways. The x-axis shows the enrichment factor; the y-axis corresponds to KEGG Pathway

To complement the transcriptomic analyses, iTRAQ LC–MS/MS was employed to examine the proteomic response of *G. kobayashii* to 4.0 mg/L (EC_100_) ARG treatment. The mass spectrometry proteomics data have been deposited to the ProteomeXchange Consortium via the PRIDE partner repository (https://www.ebi.ac.uk/pride) with the dataset identifier PXD010913. A total of 2850 proteins were identified on the basis of 285,002 highly confident spectra, of which 11,540 were peptides. A *p-*value of less than 0.05 and a 1.2-fold change of abundance were used to identify proteins that were differentially expressed between the treated and control group. 335 unique proteins (218 up and 117 down-regulated) were found to have significantly changed in abundance when *G. kobayashii* were treated with 4 mg/L ARG for 0.5 h (Fig. 8c). It's worth noting that the expression of three myosin related proteins was also altered (Fig. 8b); myosin heavy chain (log_2_FC of − 0.34), myosin regulatory light chain (log_2_FC of 0.53), and myosin heavy chain non muscle (log_2_FC of − 0.31). However, the abundance of UNC-89 at protein level displayed no obvious distinction by ARG treatment (FC = 1.15, *p *value = 0.07). Figure 8c showed the top 20 significantly enriched KEGG pathways of the DEPs, including “regulation of actin cytoskeleton” and “focal adhesion”.

## Discussion

Gold nanoparticles are arguably the most versatile nanomaterials reported up to now [45]. Due to the biological and chemical properties, their wide applications range from drug delivery to cancer therapy [46–48]. In the present study, gold nanoprobes were employed to identify drug targets in parasites for the first time. GNPs with ARG on the surface can enter live parasites and hold their antiparasitic activity. By interrogating the cellular proteome in parasite lysate, myosin-2 and UNC-89 were identified as the novel direct target proteins of ARG in *G. kobayashii*. Furthermore, results of iTRAQ quantitative proteomics and RNA-seq analyses indicated that myosin-2 expressions were down-regulated both in transcript and protein levels, but for UNC-89, only in mRNA level.

For thousands of years, nature has provided us with a rich source of bioactive molecules with medicinal property to treat many diseases [49, 50]. Numerous compounds display desirable drug efficacy and undesirable toxic responses in parallel, identifying their molecular targets will provide valuable information for thoroughly exploiting the therapeutic potential and minimizing the adverse side effects [50]. The most widely used approach currently to identify drug targets utilizes affinity purification coupled with mass spectrometry (MS) [51]. In contrast to the large size of traditional solid support, GNPs with ligands on the surface showed the ability to enter the cell to contact directly with target proteins. Here, GNP-**6** were found to be homogenously distributed over the cytoplasm and concentrated to some extent at the nucleus in the highest concentration group (Fig. 4). In accordance with the previous studies, several GNPs appeared in the area of the nucleus of HeLa, MCF-7, CBE, and HepG2 cells [52, 53], besides, GNPs were also found inside the mitochondrion [53]. Although larger GNPs have been considered to be mainly located in the endosome [54–56], small particles possessed the ability to enter anywhere inside of the cells. These findings suggested that GNPs as one of emerging solid supports could be used for identification of drug target proteins even located in the nucleus or mitochondrion.

Although many applications of GNPs have been reported, there have only been a few reports on their usage in drug target identification [8]. In this study, GNPs were demonstrated suitable for determining drug target proteins in parasite, UNC-89 and myosin-2 were identified as the potential direct target proteins of ARG in *G. kobayashii*. UNC-89 is a giant polypeptide located at the muscle, the human homologue is obscurin [57]. Loss-of function *unc-89* mutants display disorganized myosin thick filaments and reduced motility [58]. Besides, myosin-2 is mainly expressed at muscle fibrils in the parasite tegument and the function is to provide motile force through ATP hydrolysis [59]. Therefore, inhibition of myosin-2 or UNC-89 in parasites results in rapid declines in motility, which are in agreement with our previous observations that bath treatment with ARG resulted in rapid declines of *G. kobayashii* motility [24]. Besides, transcriptomic and iTRAQ proteomic analyses were further adopted to investigate the global responses of *G. kobayashii* against ARG treatment. The transcripts of myosin-2 and UNC-89 displayed obvious downregulation whereas only myosin-2 showed same tendency in protein level. These data indicated that myosin-2 may be considered as the promising drug target of ARG against *Gyrodactylus*. Further studies will characterize the ARG binding site of myosin-2 and the influence to parasite survival in the host.

In summary, gold nanoprobes have been used to identify targets for ARG in fish parasites by first validating its antiparasitic activity and then interrogating the proteome in parasite lysate. iTRAQ proteomics and RNA-seq analyses further validate the results. Our findings provided a new strategy for the target identification of antiparasitic drugs and demonstrated the power of GNPs in drug discovery research.

## Supplementary information

**Additional file 1.** Supplementary information about compounds synthesis, purification, characteristics as well as GNPs synthesis.

**Additional file 2.** In vivo antiparasitic efficacy of compound **2** against *Gyrodactylus kobayashii*.

## Data Availability

All data generated or analyzed during this study are included in this published article.

## Abbreviations

ARG: Arctigenin
GNPs: Gold nanoparticles
UNC-89: Muscle M-line assembly protein
FTIR: Fourier transform infrared
GO: Gene Ontology
DEGs: Differentially expressed genes
KEGG: Kyoto encyclopedia of genes and genomes
iTRAQ: Isobaric tags for relative and absolute quantification
