# Supplementary material for: Proteome interrogation using gold nanoprobes to identify targets of arctigenin in fish parasites
Source: J Nanobiotechnology. 2020 Feb 18;18:32. doi: 10.1186/s12951-020-00591-9 (PMC7027246; doi:10.1186/s12951-020-00591-9)
Supplement: Supplementary file 1 — Additional file 1. Supplementary information about compounds synthesis, purification, characteristics as well as GNPs synthesis. [file 12951_2020_591_MOESM1_ESM.docx]

**Additional data**

**Proteome Interrogation Using Nanoprobes to Identify Targets of Arctigenin in Fish Parasites**

1. Chemistry

Reagents and all solvents were analytically pure grade and were used without further purification. Silica gel (GF_254_) (Qingdao Marine Chemical Factory, China) were used for thin layer chromatographic (TLC) analysis, and all of thespots and bands were detected by UV irradiation (254, 365 nm). Column chromatography (CC) was performed silica gel (200–300 mesh; Qingdao Marine Chemical Factory, China). General ^1^H and ^13^C NMR spectra were measured with a Bruker AM500 spectrometer at 500 and 126 MHz. The chemical shifts are expressed in parts per million (δ value) downﬁeld from tetramethylsilane, using tetramethylsilane (TMS) (δ = 0) and/or residual solvents such as dimethyl sulfoxide (DMSO) (δ = 2.50) as an internal standard. High resolution ESI-MS data were obtained on an AQ‐Exactive mass spectrometer (Thermo Fisher Scientific).

*1.1 The procedure for the synthesis of* ***compound*** *2*

**Fig. S1.** Synthesis of compound **2**

To a stirred solution of ARG (6.74 g, 18 mmol) and NaBH_4_ (0.66 g, 18 mmol) in 50 mL dry THF was added BF_3_·E_2_O (2.5 mL, 21.6 mmol) at 0℃ slowly. Then the mixture was stirred at room temperature for 3 h. After completion of the reaction, 20 mL saturated NaHCO_3_ were added, and the solution was extracted with ethylene acetate (3 × 200 mL). The combined organic extracts were washed with brine, dried, filtered, and concentrated under reduced pressure. The crude product was dissolved in 50 mL dichloromethane, and *p*-TsOH (20 mg) was added to the solution. The mixture was stirred under reflux for 1 h, after completion of the reaction, the solution was extracted with ethylene acetate (3 × 200 mL). The organic layer was combined, dried with anhydrous Na_2_SO_4_, and concentrated under reduced pressure. The crude product was purified via silica gel column chromatography with mixed petroleum ether and ethyl acetate (3:1, v/v) as eluent, and resulted in a white solid.

Compound 2: ^1^H NMR (500 MHz, CDCl_3_) δ 6.80 (d, *J* = 7.8 Hz, 1H), 6.76 (d, *J* = 8.0 Hz, 1H), 6.67 (d, *J* = 8.0 Hz, 1H), 6.62 (s, 2H), 6.60 (s, 1H), 5.63 (s, 1H), 3.84 (d, *J* = 7.1 Hz, 3H), 3.80 (s, 6H), 3.54 (d, *J* = 11.1 Hz, 2H), 3.23 (s, 2H), 2.79 – 2.60 (m, 4H), 1.86 (s, 2H). ^13^C NMR (126 MHz, CDCl_3_) δ 148.99, 147.41, 146.60, 143.97, 133.30, 132.55, 121.78, 121.16, 114.29, 112.27, 111.58, 111.26, 60.85, 56.04, 55.96, 55.94, 44.07, 43.95, 36.04, 35.97. ESI-MS: m/z 399 (M- Na).

*1.2. Synthesis of compound 6 and 10*

**Fig. S2.** Synthesis of compound **6**

General procedure for synthesis of compound 3

To a stirred solution of compound **1** (5.58 g, 15 mmol) in 150 mL acetone were added anhydrous K_2_CO_3_ (5 g, 36 mmol) at room temperature. After stirring at room temperature for 30 min, 1,6-dibromohexane (10.98 g, 45 mmol) was added to the reaction mixture, and whole was refluxed at 70°C for 24 h. The precipitate was filtered off and washed with acetone (4×100 mL). The solvent was evaporated under reduced pressure, and the residue was treated with water (200 mL) and extracted with dichloromethane (4×150 mL). The organic layer was combined, dried with anhydrous Na_2_SO_4_, and concentrated under reduced pressure. The crude product was purified via silica gel column chromatography with mixed petroleum ether and ethyl acetate (1:1, v/v) as eluent to achieve compound **3**. ^1^H NMR (500 MHz, CDCl_3_) δ 6.75 (dd, *J* = 9.9, 8.3 Hz, 2H), 6.68 (s, 1H), 6.63 (dd, *J* = 8.1, 1.6 Hz, 1H), 6.54 (dd, *J* = 8.1, 1.5 Hz, 1H), 6.48 (d, *J* = 1.5 Hz, 1H), 4.12 – 4.07 (m, 1H), 3.97 (t, *J* = 6.6 Hz, 2H), 3.86 (d, *J* = 10.3 Hz, 1H), 3.84 (s, 3H), 3.81 (d, *J* = 3.5 Hz, 6H), 3.40 (t, *J* = 6.8 Hz, 2H), 2.93 (qd, *J* = 14.1, 6.0 Hz, 2H), 2.66 – 2.45 (m, 4H), 1.95 – 1.76 (m, 4H), 1.54 – 1.44 (m, 4H). ^13^C NMR (126 MHz, CDCl_3_) δ 178.79, 149.56, 149.08, 147.91, 147.48, 130.56, 130.38, 121.46, 120.63, 112.98, 112.90, 111.91, 111.39, 71.30, 68.88, 56.04, 55.98, 55.92, 46.62, 41.19, 38.23, 34.58, 33.90, 32.72, 29.11, 27.99, 25.31. ESI-MS: m/z 557 (M-Na).

General procedure for synthesis of compound 4

Compound **4** (6.16 mmol, 3.3 g), phthalimide potassium (18.89 mmol, 3.5 g) were dissolved in 100 mL dimethylformamide (DMF). The reaction mixture was refluxed at 100°C for 24 h. The mixture was cooled to room temperature, filtered, washed with water (1000 mL), and filtered under negative-pressure, and dried to produce the compound 4 at high purity and in high yield. This compound was used directly without further purification. 1H NMR (500 MHz, CDCl3) δ 7.83 (dd, J = 5.4, 3.1 Hz, 2H), 7.70 (dd, J = 5.4, 3.0 Hz, 2H), 6.75 (dd, J = 7.9, 6.5 Hz, 2H), 6.67 (d, J = 1.5 Hz, 1H), 6.54 (dd, J = 8.1, 1.6 Hz, 1H), 6.48 (d, J = 1.5 Hz, 1H), 4.10 (dd, J = 8.9, 7.0 Hz, 1H), 3.96 (t, J = 6.7 Hz, 2H), 3.86 (s, 1H), 3.84 (s, 3H), 3.82 (s, 3H), 3.80 (s, 3H), 3.68 (t, J = 7.2 Hz, 2H), 2.96 – 2.87 (m, 2H), 2.66 – 2.44 (m, 4H), 1.85 – 1.77 (m, 2H), 1.70 (dt, J = 14.9, 7.5 Hz, 2H), 1.54 – 1.46 (m, 2H), 1.41 (dt, J = 14.9, 7.3 Hz, 2H). 13C NMR (126 MHz, CDCl3) δ 178.86, 168.58, 149.66, 149.17, 148.00, 147.62, 134.00, 132.29, 130.62, 130.37, 123.30, 121.52, 120.70, 113.08, 113.00, 112.03, 111.51, 71.35, 69.00, 56.11, 56.05, 55.99, 46.71, 41.23, 38.32, 38.04, 34.68, 29.23, 28.66, 26.77, 25.74. ESI-MS: m/z 624 (M-Na).

General procedure for synthesis of compound 5

Excessive hydrazine hydrate (10 mL) was added to a magnetically stirred solution of compound **4** (2.7 g, 4.49 mmol) in 100mL ethanol. The reaction was allowed to carry out at 80 ºC for 6 h. When the reaction was completed, the precipitate was removed by filtration, and the filtrate was distilled under reduced pressure to remove ethanol. The residue was treated with water (150 mL) and extracted with chloroform (3×90 mL). The organic layer was combined, dried with anhydrous Na_2_SO_4_, and concentrated under reduced pressure to achieved compound **5**. The product was directly used in the next step without further purification.

General procedure for synthesis of compound 6

Lipoic acid (1.9 mmol, 0.387 g), N, N'-dicyclohexylcarbodiimide (DCC, 1.9 mmol, 0.368 g), 4-dimethylaminopyridine (DMAP, 1.0 mmol, 0.12 g), compound **5** (1.5 mmol, 0.75 g) were mixed in 20 mL dichloromethane. The reaction mixture was stirred at room temperature for 24 h as monitored by TLC. After the reaction was completed, the precipitate was removed by filtration, and the filtrate was distilled under reduced pressure to remove dichloromethane. The residue was purified by column chromatography, eluting with dichloromethane/methanol (1:1) to achieve compound **6**. 1H NMR (500 MHz, CDCl3) δ 6.75 (t, J = 6.5 Hz, 2H), 6.67 (s, 1H), 6.63 (d, J = 7.9 Hz, 1H), 6.54 (d, J = 8.0 Hz, 1H), 6.48 (s, 1H), 5.52 (s, 1H), 4.10 (t, J = 7.5 Hz, 1H), 3.96 (t, J = 6.3 Hz, 2H), 3.87 (d, J = 6.3 Hz, 1H), 3.84 (s, 3H), 3.81 (d, J = 4.1 Hz, 6H), 3.26 – 3.20 (m, 2H), 3.20 – 3.04 (m, 2H), 2.98 – 2.86 (m, 2H), 2.66 – 2.39 (m, 5H), 2.15 (t, J = 7.1 Hz, 2H), 1.93 – 1.78 (m, 3H), 1.71 – 1.59 (m, 4H), 1.55 – 1.34 (m, 9H). 13C NMR (126 MHz, CDCl3) δ 178.85, 172.74, 149.53, 149.09, 147.93, 147.52, 130.57, 130.34, 121.51, 120.67, 112.92, 111.93, 111.41, 71.34, 68.92, 56.55, 56.07, 56.01, 55.95, 46.66, 41.21, 40.35, 39.49, 38.57, 38.26, 36.63, 34.72, 34.61, 29.67, 29.18, 29.01, 26.75, 25.81, 25.55. ESI-MS: m/z 682 (M-Na).


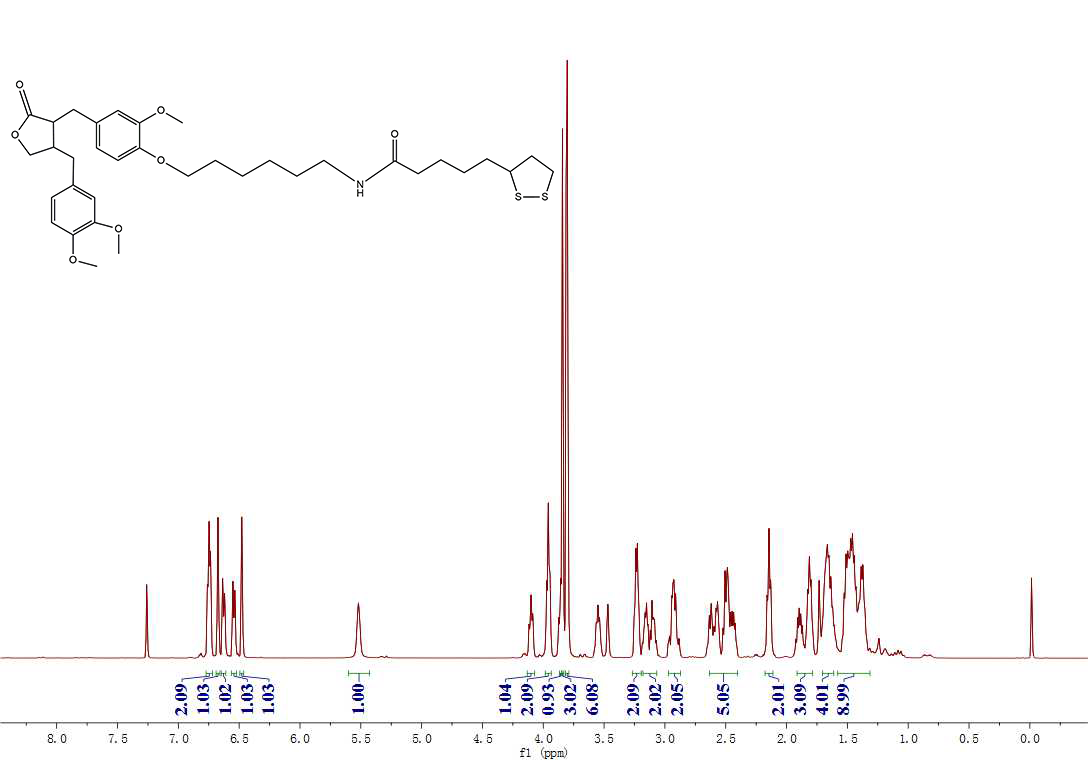


**Fig. S3.** ^1^H NMR spectrum of compound **6**

**Fig. S4.** ^1^H NMR spectrum of compound **6**

**Fig. S5.** Synthesis of compound **10**

Compound **10** was prepared according to the synthetic route of compound **6**.

Compound 10: 1H NMR (500 MHz, DMSO) δ 7.71 (t, J = 5.5 Hz, 1H), 6.75 (dd, J = 8.0, 5.5 Hz, 2H), 6.60 (s, 2H), 6.56 (t, J = 9.0 Hz, 2H), 4.52 (s, 2H), 3.83 (t, J = 6.5 Hz, 2H), 3.66 (s, 3H), 3.62 (s, 6H), 3.18 – 3.00 (m, 4H), 2.98 (dd, J = 6.5, 6 Hz, 2H), 2.50 – 2.45 (m, 5H), 2.35 (td, J = 12.5, 6.5 Hz, 2H), 2.01 (t, J = 7.5 Hz, 2H), 1.88 – 1.73 (m, 4H), 1.67 – 1.56 (m, 4H), 1.51 – 1.43 (m, 4H), 1.38 – 1.29 (m, 6H). 13C NMR (126 MHz, DMSO) δ 171.79, 148.79, 148.49, 146.79, 146.16, 134.03, 133.95, 120.95, 120.87, 113.07, 112.94, 112.66, 111.66, 68.27, 60.26, 56.19, 56.13, 55.51, 55.38, 55.29, 42.47, 39.93, 38.38, 38.11, 35.27, 34.14, 34.01, 33.53, 29.16, 28.88, 28.34, 28.24, 26.22, 25.34, 25.13, 24.30. ESI-MS: m/z 686 (M-Na).


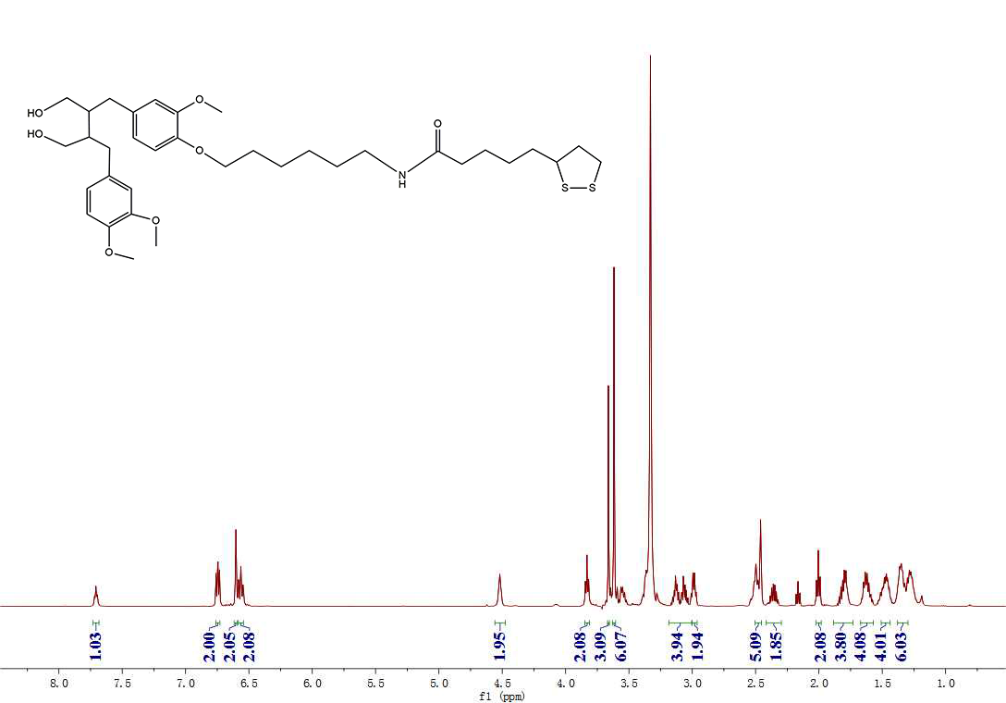


**Fig. S6.** ^1^H NMR spectrum of compound **10**

**Fig. S7.** HRMS spectrum of compound **10**

2. Synthesis of GNP-6 and GNP-10

10.8 mL of water containing hydrogen tetrachloroaurate (III) tetrahydrate (132.4 mg, 0.032 mmol) was added to a solution of compound **6** or **10** (0.216 mmol) in DMF (100.0 mL). After stirring for 30 min at room temperature, sodium tetrahydroborate (34.2 mg, 0.928 mmol) in 58.8 mL water was added to the mixture dropwise. The solution turned red immediately and was stirred for another 4 h at room temperature. 1 M HCl was added to the reaction mixture dropwise to neutralize the excess sodium tetrahydroborate until the pH reached 7.0. The reaction mixture was then centrifuged at 15000 rpm for 15 min. The supernatant was decanted and the solid was dissolved in 10 mL methanol and deionized water alternatively by sonication and centrifuged again at 15000 rpm for 15 min. This wash-centrifugation cycle was repeated five times. After the final washing step, the nanoparticles were dried in vacuum at 50ºC for 12 h.
