# Supplementary material for: Proteome interrogation using gold nanoprobes to identify targets of arctigenin in fish parasites
Source: J Nanobiotechnology. 2020 Feb 18;18:32. doi: 10.1186/s12951-020-00591-9 (PMC7027246; doi:10.1186/s12951-020-00591-9)
Supplement: Supplementary file 2 — Additional file 2. In vivo antiparasitic efficacy of compound 2 against Gyrodactylus kobayashii. [file 12951_2020_591_MOESM2_ESM.docx]

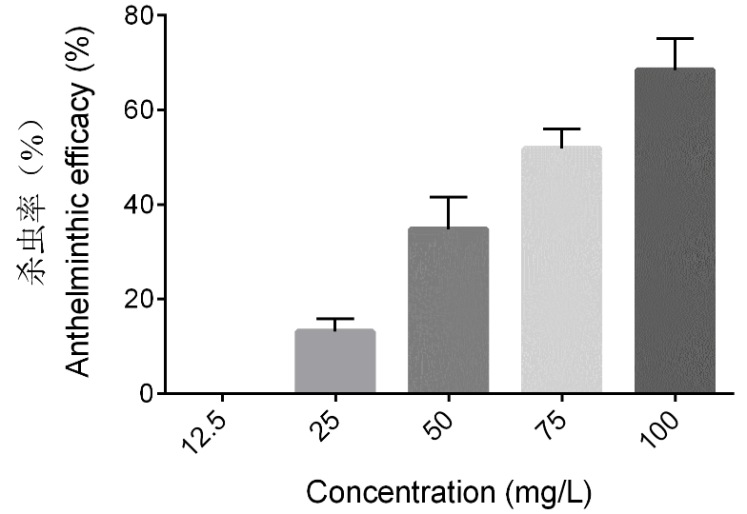


**Fig. S8** *In vivo* antiparasitic efficacy of compound **2** against *Gyrodactylus kobayashii* after 24 of exposure. Values are means ± SD (standard deviation) of 3 replicates.
